# Challenges in measuring individual differences of brain function

**DOI:** 10.1162/imag_a_00430

**Published:** 2025-01-07

**Authors:** Ting Xu, Gregory Kiar, Xi-Nian Zuo, Joshua T. Vogelstein, Michael P. Milham

**Affiliations:** Child Mind Institute, Center for the Integrative Developmental Neuroscience, New York, NY, United States; Developmental Population Neuroscience Research Center, IDG/McGovern Institute for Brain Research, State Key Laboratory of Cognitive Neuroscience and Learning, Beijing Normal University, Beijing, China; John Hopkins University, Baltimore, MD, United States; Nathan Kline Institute for Psychiatric Research, Orangeburg, NY, United States

**Keywords:** individual differences, intraindividual variation, brain function, reliability, fingerprinting

## Abstract

With a growing interest in personalized medicine, functional neuroimaging research has recently shifted focus from the evaluation of group-level summaries to associating individual differences in brain function with behaviors. However, this new focus brings forth challenges related to accurately measuring the sources of individual variation in functional signals. In this perspective, we highlight the impact of within-individual variations and discuss the concept of measurement reliability as a critical tool for accounting for within- and between-individual variations when measuring individual differences in brain function.

*“No man ever steps in the same river twice” - Heraclitus.*

The human brain is a dynamic system, continuously updating itself in response to internally and externally generated stimuli and task demands in support of higher order cognition (Fox et al., 2005). From moment to moment, patterns of neural activity traverse the structural connectome, like waves moving along a riverbed that is being remodeled over time. This notion is reflected in classical (psychometric) test theory, which asserts that no two points in time are ever exactly the same for an individual—a reality that challenges efforts seeking to consistently and meaningfully measure brain function (e.g., task activation), particularly those aiming to understand differences among individuals (DeVellis, 2006;Seghier & Price, 2018). Any experimental measurement merely captures a snapshot of the function and behavior produced by an individual brain at a given moment. Arrays of measurements can be combined to test hypotheses across time scales (e.g., milliseconds, seconds, minutes, days, months, years) that are relevant to answering scientific questions. Considered in the present context, variation across measurements is the rule in the evaluation of brain function for individuals—not the exception. However, in the midst of this change is a dynamic equilibrium supported by homeostatic processes—while our brains and behaviors evolve, we remain, to an extent, the same individuals. It is this equilibrium that allows the brain to maintain order despite its dynamic nature. And, it is this equilibrium that makes it feasible for measurements of trait differences in brain function (e.g., functional connectivity) to approach repeatability over time (i.e., agreement between temporally independent test results), despite never being exactly the same (Gratton et al., 2018;Laumann et al., 2017;Poldrack et al., 2015;Yang et al., 2020).

This*Perspective*illustrates the impact of within-individual variations when measuring individual differences. In addition, we delineate challenges inherent to the measurement of trait differences in brain function that must be addressed for individual difference research to reach clinical and scientific utility. The challenges discussed are applicable regardless of whether the trait measures of brain function are attempting to capture the central tendency (e.g., static functional connectivity matrices) or the variation (e.g., time-varying properties for functional connectivity patterns) observed in individuals.

## 1 Within-Individual Variation Matters in Detecting Differences Between Individuals

**Fig. 1.**
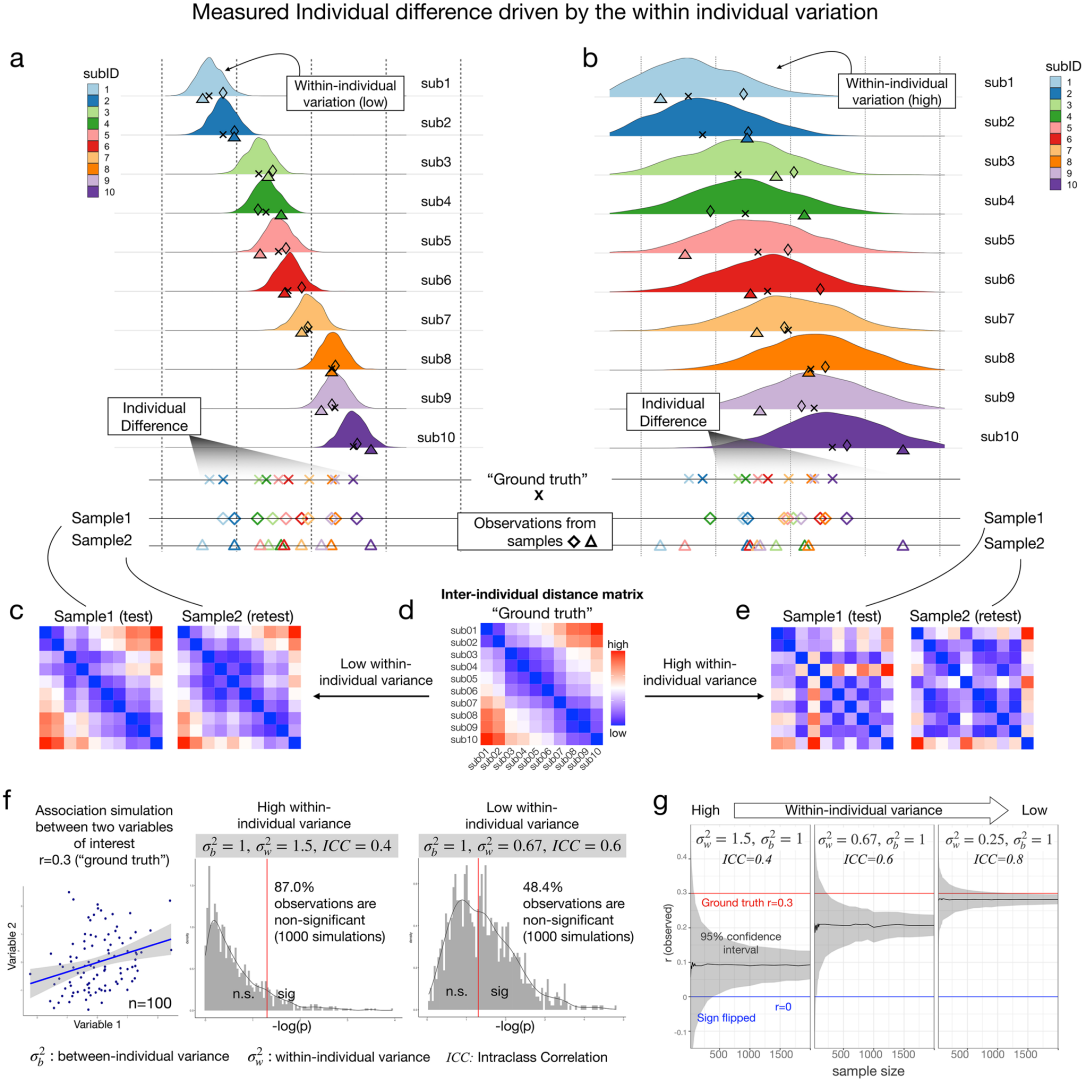
Simulations demonstrating the effects of within-subject variation on measuring individual differences. The observed individual differences can vary due to the within-subject variation. Simulations of observed individual differences from 10 individual subjects are generated from the same known “ground truth” (marked in cross “X”). (a–b) Simulations of a single variable of interest, which were scored repeated twice (sample1: diamond, sample 2: triangles) from these 10 individual subjects with a low (a) and high (b) within-subject variation. (c-e) Interindividual distance matrices of “ground truth” (d) and samples (c, e). (f) Simulations of correlations between two variables of interest (ground truth r = 0.3). The observed correlations between two variables (e.g. thickness and IQ) were calculated within each simulation and they vary due to the within-subject variations (sample size = 100, 10,000 simulations). The greater within-subject variation introduces noisier samples and more nonsignificant results. (g) Simulations of correlations between two variables of interest (ground truth r = 0.3), conducted with varying sample sizes and levels of within-subject variations (10,000 simulations per sample size). A large sample is required to detect the association between variables of interest when individual within-subject variation is sufficiently high.

If one is attempting to obtain a stable estimate from a dynamic system, multiple measurements are required to estimate the central tendency. However, most studies are cross-sectional without repeated measurements from the same individuals. As a result, within-individual variation is often overlooked or misinterpreted as interindividual variation when studying individual differences in brain function. We demonstrate this by simulating an illustrative example in which individuals with known “ground truth” interindividual differences can generate distinct observed individual differences, driven by within-individual variation. First, we simulated a “ground truth” score for each of the 10 individual subjects (marked in cross “X”Fig. 1a-b), which is the expected value of an individual score distribution. In an ideal scenario, we would obtain the true individual differences (i.e., interindividual distance matrix,Fig. 1d). However, due to the measurement variability within each subject, the observed scores from each measurement instance (sample 1 vs. sample 2 inFig. 1a-b) vary, resulting in divergent observed individual differences (Fig. 1c-e). As within-subject measurement variation increases (e.g., more variation between measurements,Fig. 1b), the observed individual differences vary even more (Fig. 1e, sample 1 vs. sample 2) and are likely be diverge further from the “ground truth”.

Importantly, such variation of brain measurements within subjects also has an impact on studies of brain-wide associations (e.g., thickness and IQ). InFigure 1f-g, we simulated repeated measures for two variables from same individuals and showed varied observed correlation scores purely driven by the variations of measures within each sampling individual. The observed correlation between two variables (e.g., thickness and IQ) based on the ground truth (r = 0.3 in simulations) can either result in (1) inconsistencies in findings between samples (i.e., insignificant or significant with a relatively reasonable sample size N = 100 inFig. 1f) or (2) lower the statistical power and require a larger sample to detect the brain-wise association when individual within-subject variation is sufficiently high (Fig. 1g, N = 20–2000). Thus, although the “ground truth” remains the same, the observed between-individual differences can be different due to within-individual variation alone, thereby impacting the power to detect brain-wise associations (Hsu et al., 2022;Zuo et al., 2019).

## 2 Sources of Variation in the Measure of Individual Differences

**Fig. 2.**
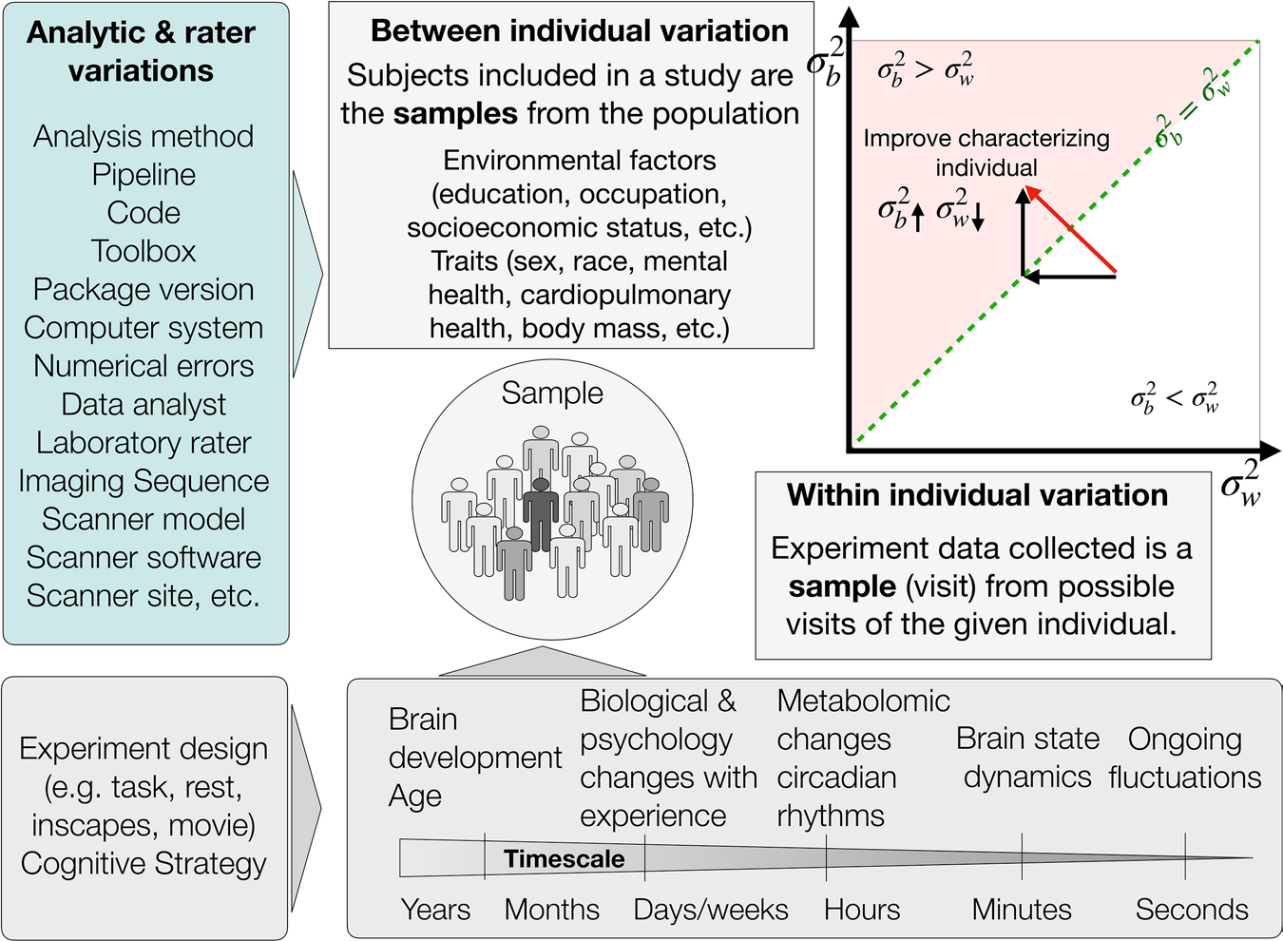
Factors affect the measure of individual differences.

Depending on the time scale of the data, sources of within-individual variation can exist between multiple levels: from moment to moment, day to day, month to month, or year to year. At each time scale, brain function can also vary with internal and external factors.Figure 2(lower panel) summarizes common potential sources of variation in brain function that can arise, whether measuring the same individual on multiple occasions, or different individuals. From moment to moment, brain functional organization varies along with ongoing fluctuation for each individual (Garrett et al., 2013). Such dynamic activity reflects the changes of the brain state with internal awareness and/or external stimulus. For example, the brain state tends to change as the mind wanders during a resting-state scan, while an individual watching a movie or undertaking a specific task (e.g., Wisconsin Card Sorting Test) will undergo state changes consistent with the ongoing stimulus (Smallwood & Schooler, 2015;Vatansever et al., 2017). On the scale of hours, diurnal rhythms during the day can alter the brain state, as well as circadian rhythms which govern the homeostatic metabolomic and have shown daily variations on functional connectomes (Hodkinson et al., 2014). These can be further affected by external factors (e.g., feeding, caffeination, sleep quality the day before the scan) (Blautzik et al., 2013;Poldrack et al., 2015). From weeks to months, seasonal effects and life events might change the individuals’ mood and cognitive functions, affecting the brain state during measurement (Di et al., 2021;Shine et al., 2016). Biological and psychological changes with individual unique experiences also lead to variations in functional organization (Poldrack et al., 2015). From year to year, age effects of brain development combined with long-term environmental–social influences (e.g., education, income) contribute to the variation in the measurement and comparison of individuals (Bethlehem et al., 2022;Hackman & Farah, 2009;Kolb et al., 1998;Tooley et al., 2021).

Regardless of the time scale one considers (e.g., seconds, minutes, days, months, years), numerous potential sources of within-individual variation are capable of individually or jointly impacting brain function and/or its measurements. The breadth of sources of variation can be overwhelming, though consistent with prior suggestions, they can be categorized into subgroups, such as acquisition-, experimental-, environmental-, participant-, and analysis-related factors (Botvinik-Nezer et al., 2020;Nichols et al., 2017;Schilling et al., 2021;Yan et al., 2013). Beyond these sources, measurement errors are additional, pervasive contributors to sampling variability, affecting both intra- and interindividual variations. Such variations may stem from fluctuations in attention or minor disruptions during testing. It is worth noting that some factors can create opportunities for understanding the impact of biologically meaningful processes on brain function (e.g., time of day) when explicitly considered in the experimental design or analysis (e.g., the MyConnectome project) (Laumann et al., 2017;Poldrack et al., 2015). However, most of these factors are a nuisance, creating undesirable perturbations in analysis and leading to potential biases in measuring individual differences, especially when the factors are systematic in nature (Glatard et al., 2015;Kiar et al., 2021). When the data are available, modeling the factors that may contribute to the intraindividual variation as covariates at the individual level is advisable.

In addition to the within-individual variation, there are diverse sources of variation in measuring individual differences of brain function (Fig. 2). The criteria and strategies employed for data collection play a crucial role in determining the extent to which the sample reflects the population of interest. This variability in recruitment methods (e.g. college, online, or community recruitment) may introduce unwanted between-individual variations into the experiments (Conley et al., 2021). Additionally, analytic variations such as pipeline, computational system, and batch effects (Fig. 2green box) may also lead to contaminated estimates of individual differences (Botvinik-Nezer et al., 2020;Haddad et al., 2023;Hedges et al., 2022;Hu et al., 2023;Kiar et al., 2021;Li et al., 2024). While recent attention has predominantly been on reproducibility of analytic variations, few studies have investigated how different analytic perturbations contribute to both within- and between-individual variations.

How do the different sources of variation impact the measurement of individual differences? In general, measurements that have relatively lower within-individual variation and higher between-individual variation lead to improved individual differentiation (Finn et al., 2017). It is important to note that neither the within- nor between-individual variation alone determines the differences observed among individuals (Zuo et al., 2019). For example, there is always variation from session to session within each individual. However, when the functional connectivity of a given individual is relatively more similar from one session to the next as compared with other individuals, this individual can be recognized and differentiated from others. When measuring individual differences of the functional connectome on a finer time scale (e.g., functional dynamics in minutes or seconds), the dynamic changes from moment to moment may not be stable within individuals. Yet, the dynamic characteristics (e.g., the principle that governs the dynamics, how the network configurations vary, etc.) may be relatively similar within individuals as compared with between individuals (Yang et al., 2020). Thus, it is important to decipher sources of variation—within- versus between-individual variations—as well as their proportional impact on overall variations, to improve individual differentiation (Gratton et al., 2018).

In neuroimaging studies, participants included in the analysis can be scanned at different times during the day, or undertake different experiment states (e.g., rest, task, movie) (Blautzik et al., 2013;Vanderwal et al., 2017). Even for the same task, participants can adopt different cognitive strategies resulting in variation between individuals (Seghier & Price, 2018). Although previous studies have demonstrated that functional networks are largely stable within individuals, that is, variation between sessions or tasks contributes less compared with between-individual variations (Gratton et al., 2018), few studies differentiate within-individual variation from total observed variation. In particular, in cross-sectional studies, an individual’s connectome is often assumed to be stable and interpreted as part of the between-individual difference (Hsu et al., 2022). This is problematic because as shown inFigure 1, within-individual variation can jeopardize the estimation of true inter-individual differences when it is large relative to between-individual differences. Such contaminations of between-individual differences from within-individual differences can compromise brain–behavior association discovery.

## 3 Quantifying the Impact of Within- and Between-Subject Variation on Measuring Individual Differences: From Fingerprinting to Reliability

A critical prerequisite for individual difference research is that variation observed between individuals should not be assumed to reflect true individual differences without accounting for the underlying variation within individuals. In test theory, it is common practice for summarizing the relative contributions of the two dimensions in a single value, namely measurement reliability. For example, when looking at continuous measurements, the ratio of between-individual variation divided by the sum of within-individual and between-individual variation, namely the intraclass correlation (ICC), is widely used to quantify how well the measure can characterize reliable individual differences (Brandmaier et al., 2018;Chen et al., 2018;McGraw & Wong, 1996). However, as modern neuroscience has increased the dimensionality of characterizations for an individual, the field has faced the challenge of how to achieve such indices for multivariate profiles (Bridgeford et al., 2021;Yoo et al., 2019). One solution that has emerged in neuroimaging is fingerprinting (i.e. identification rate), a nonparametric index that quantifies whether the individuals can be matched with themselves across repetitions (Finn et al., 2015). Alternatively, three approaches to generalizing the ICC to multivariate formulations have emerged, including (i) a parametric extension of the classic ICC formulation, image intraclass correlation coefficient (I2C2) (Shou et al., 2013), (ii) distance-based ICC (dbICC), which reformulates the ICC in terms of distances (M. Xu et al., 2021), and (iii) discriminability, a nonparametric index that assesses the degree to which an individual’s repetitions are relatively similar to one another (Bridgeford et al., 2021;Wang et al., 2020).

With many reliability indices emerging to take on the challenge of measuring individual differences and calculating reliability, understanding the advantages and limitations of each can aid in selecting the appropriate measure for a given application. First, we draw attention to parametric assumptions for a given data set (e.g., Gaussian distribution, homogeneous variance). In cases where data are not Gaussian distributed, ICC and dbICC can be misleading. Additionally, in some cases, which often occur in neuroimaging studies, ICC and dbICC are negative due to the negative difference between two mean-square terms in the computational formula (Chen et al., 2018); although not inherently problematic, in practice, negative ICC/dbICC is not interpretable, and can be avoided in some more recent ICC formulations (e.g., the Restricted Maximum Likelihood method). Second, each index provides different sensitivity. High discriminability is required for fingerprinting (i.e., identification), but not vice versa; in some conditions, fingerprinting and discriminability will diverge, with fingerprinting potentially leading to the wrong conclusions (Milham et al., 2021). Previously we illustrated such situation that the individual difference is relatively discriminable, but the fingerprinting score is zero—and thus, fingerprinting, may mislead its users with respect to the potential for optimization and eventual usage (Supplementary MaterialsandMilham et al., 2021).

An important caveat to be aware of when using multivariate indices of reliability is that they do not guarantee the reliability of each univariate feature. It is well established that reliability differs between regions and connections in the brain (Jiang et al., 2023;Noble et al., 2019). Some features may contribute more to the detectability of individual differences than others (Hong et al., 2020), and it is possible that some features may differentiate a subset of individuals, but not all. As such, the reliability for a multivariate profile should not be assumed to reflect that for its individual features. A sensitivity test (e.g., leave-one-out analysis) to examine the contributions for each of the individual features or the univariate reliability should be considered.

Additionally, while reliability is a prerequisite of measurements of interest, it is not sufficient for establishing validity or implying predictive power for other measurements. Reliability ensures that a brain or behavioral feature can be consistently measured within an individual across specific time scales. However, consistency alone does not inherently validate the feature as an accurate representation of the intended construct. A reliable feature may still lack relevance or utility in explaining individual differences in behavior or predicting specific outcomes. This raises a question: why not focus exclusively on predictive power in brain–behavior association studies, disregarding reliability? The answer lies in the fact that the confidence in observed associations between two measurements (x and y) is impacted by their reliability, as expressed by the equation$r_{true}\left(x,y\right)=r_{observed}\left(x,y\right)\sqrt{reliability_{x}\times reliability_{y}}$(Nikolaidis et al., 2022). High reliability strengthens confidence in the true relationship between variables of interest. Conversely, in cases of low predictive accuracy, knowing the reliability of the measurements can help determine whether the weak prediction reflects a truly low relationship or a possible artifact of measurement error and sample variability. Therefore, establishing both reliability and validity is imperative as this distinction is crucial for interpreting findings for understanding individual differences in biomarker discovery.

## 4 Moving Forward

When considering the above discussed challenges, it becomes evident that differentiation of within-individual from the observed between-individual variances is crucial. Toward this end, studies of individual differences in the brain and behavior would benefit from the inclusion of repeat assessment in their design, at least for a subportion of collected samples, to allow for assessment of within-individual variation. Previous studies have highlighted that increasing number of repetitions and scan duration enhances reliability (Noble et al., 2017), Notably, acquiring and concatenating multiple shorter scans have shown a clear advantage over a single long scan within individuals for improving reliability (Cho et al., 2021). The sample size and scan time within individuals appear to be broadly interchangeable for total quantities up to 20–30 min per individual in brain–behavior prediction. The prediction accuracy improves as the total amount of data, following a logarithmic model (Ooi et al., 2024). Additionally, studies that assess the variation across analytic methods and experiment protocols should also be encouraged to understand the contributions to sources of variation at both within- and between-individual levels. In addition, the changes of the brain at different time scales and the variation of participant samples (e.g. education, socioeconomic status, etc.) need to be considered (e.g. included in the statistic model) in studies of individual differences. While there is an increasing focus on the standardization of measurements and procedures, enabling centralized assessments for researchers using standardized protocols, the feasibility and generalizability of this approach across population characteristics need to be established. This requires careful consideration to ensure that standardized approaches are applicable and meaningful across diverse participant demographics.

Regarding the quantification of individual differences and its source of variation, we posit that standardizing methods for assessing reliability is as important as standardization of measurement protocols—a central focus of the past decade of work in the imaging community. We have recently developed ReX (Reliability eXplorer) as a tool for rapidly calculating, visualizing, and interrogating these variances, though advanced consideration of data needs is critical to making such calculations possible (T. Xu et al., 2023). This is not to suggest that there can only be one measure of individual difference, but rather, that there would be the benefit of achieving consensus on the interpretations of these measures and which may be optimal for differing situations, as well as agreement on their limitations.

Overall, individual difference research is increasingly highlighting the challenges of reliable measurement for human brain function. In practice, within-individual variation is always embedded in the observed scores of interindividual differences. It is important to conceptualize reliability in terms of its component variances—particularly for studies of brain function, where the within-subject variance can never be zero, and can be noise or meaningful variation of interest depending on testing circumstances. Future work that studies sources of variations across experiments and analytic methods, as well as the changes at different time scales within individuals, can facilitate studying individual differences in brain and behavior in neuroscience and psychology.

## Data and Code Availability

The code used in the simulations forFigure 1is available on GitHub:https://github.com/TingsterX/Reliability_Explorer/tree/main/simulation.

## Author Contributions

T.X. analyzed and wrote the original draft. G.K., X.-N.Z., J.T.V., M.P.M. reviewed and edited the manuscript.

## Declaration of Competing Interest

The authors declare no competing interests

## Supplementary Material

Supplementary Material
