# Supplementary material for: Challenges in measuring individual differences of brain function
Source: Imaging Neurosci (Camb). 2025 Jan 7;3:imag_a_00430. doi: 10.1162/imag_a_00430 (PMC12319922; doi:10.1162/imag_a_00430)
Supplement: Supplementary Material [file imag_a_00430-supp.pdf]

Supplementary Materials

Fig S1 illustrates the indices in measuring individual differences. The toy example includes two repetitions in ten participants. Fig S1a shows the pairwise symmetric distance matrix between each of the repetitions, ordered by participants. The block-diagonal elements indicate how different the repeated measures are within participants, whereas off-diagonal elements represent the dissimilarity of measures between participants. We then construct a two-dimensional space by plotting the observed between-individual elements along the y-axis against the within-individual value on the x-axis (Fig S1b). Each between-individual element for a row or column in the distance matrix can be represented by an array of dots in the distance space. It's important to note that the x=y line in this space represents the case that within-individual distance equals the observed between-individual distance. The dots above the x=y line (i.e., x<y, red shaded zone) represent the cases that the difference within individuals is lower than the difference observed between individuals. By counting the number of dot-columns that fully fall above the x<y zone (i.e., within-individual distance < between-individual distance) as compared to the number of individuals (i.e., the total number of dot columns), we can calculate the percentage that the individuals can be successfully differentiated from the group (i.e., identification rate, a.k.a. fingerprinting). In the toy example, 3 out of 10 dot columns are above the x=y line and resulting in a fingerprinting score of 0.3 (Fig S1b). If more dots fall above the x<y zone, the repetitions within individuals are more likely similar to one another but distant from other individuals. Instead of counting the percentage of the dot columns above the x=y line, discriminability measures the percentage of dots above the x=y line as compared to the total number of dots. We can also estimate the ratio of between-individual variation in total observed individual variation (i.e., ICC, equations in Fig S1b).

In neuroimaging and psychology, ICC and its extension (e.g. I2C2, dbICC) are widely used approaches for assessing reliability (i.e. the degree of agreement or consistency of measures) (Noble, Scheinost, and Constable 2019; Xu, Reiss, and Cribben 2021; Shou et al. 2013; Zuo and Xing 2014). In the context of measuring individual differences, ICC, by definition, represents how much inter-individual variation can be measured. This is particularly useful in studying individual differences and brain-behavior relationships. For example, if ICC=0.5 of a given measurement, regardless of neuroimaging or behavioral measurement, indicates that the observed measurement can only capture 50% of the inter-individual variation of interest from observed inter-individual variation that is contaminated with within-individual variations.

It's worth noting that ICC or fingerprinting might fail to capture the individual differences. To understand this, we draw your attention to a simple scenario using a toy model in Fig S1. Fig S1c shows the case that ICC failed to capture the individual differences when Gaussian assumption is violated. The nonparametric method, fingerprinting can also failed to detect relatively reliable data (Fig S1d). Specifically, situations can arise where most, but not all, of the between-individual distances are larger than the within-individual distance (i.e., most dots are in the x < y zone, but none of the entire dot columns are in the x < y zone; x = within-individual distance; y = between-individual distance). In such instances, the individual difference is relatively discriminable, but the fingerprinting score would be zero — and thus, fingerprinting, may mislead its users with respect to the potential for optimization and eventual usage.

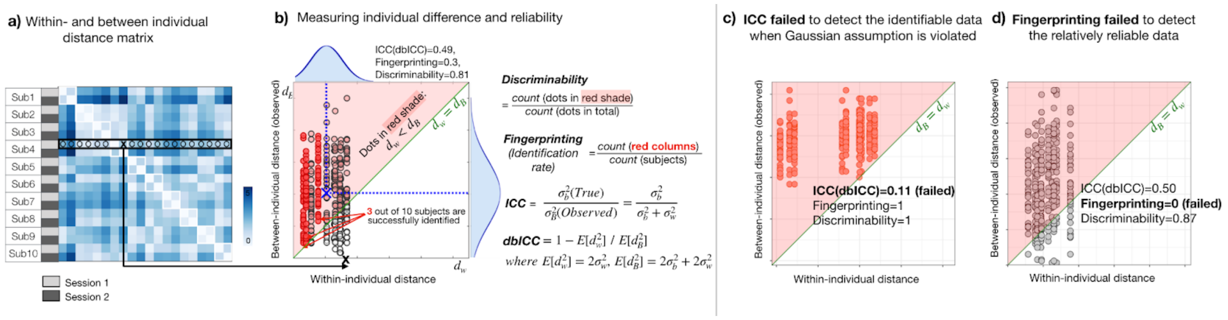

Fig S1. The two-dimensional distance field map characterizes within- and between-individual variability. a) Distance matrix between individuals and two repeated measures. Diagonal submatrices depict within-individual distances, while off-diagonal elements are between-individual distances **b)** Identifying individuals is quantified by discriminability, intraclass correlation (ICC), or fingerprinting (i.e., identification rate). Discriminability is estimated by the probability that the distance between sessions within each individual is smaller than the distance to other individuals. ICC values are estimated with the ratio of between-individual variation divided by the sum of within-individual and between-individual variation. Fingerprinting is estimated by the proportion of individuals correctly identified (modified from (Milham, Vogelstein, and Xu 2021)). **c-d)** Examples in which fingerprinting and ICC are essentially uninformative and arguably provide misleading information.

References

Milham, Michael P., Joshua Vogelstein, and Ting Xu. 2021. "Removing the Reliability Bottleneck in Functional Magnetic Resonance Imaging Research to Achieve Clinical Utility." *JAMA Psychiatry* 78 (6): 587–88.

Noble, Stephanie, Dustin Scheinost, and R. Todd Constable. 2019. "A Decade of Test-Retest Reliability of Functional Connectivity: A Systematic Review and Meta-Analysis." *NeuroImage* 203 (December): 116157.

Shou, H., A. Eloyan, S. Lee, V. Zipunnikov, A. N. Crainiceanu, N. B. Nebel, B. Caffo, M. A. Lindquist, and C. M. Crainiceanu. 2013. "Quantifying the Reliability of Image Replication Studies: The Image Intraclass Correlation Coefficient (I2C2)." *Cognitive, Affective & Behavioral Neuroscience* 13 (4): 714–24.

Xu, Meng, Philip T. Reiss, and Ivor Cribben. 2021. "Generalized Reliability Based on Distances." *Biometrics* 77 (1): 258–70.

Zuo, Xi-Nian, and Xiu-Xia Xing. 2014. "Test-Retest Reliabilities of Resting-State FMRI Measurements in Human Brain Functional Connectomics: A Systems Neuroscience Perspective." *Neuroscience and Biobehavioral Reviews* 45 (September): 100–118.
